# Supplementary material for: Proteomic Characterization of the Alzheimer’s Disease Risk Factor BIN1 Interactome
Source: Mol Cell Proteomics. 2025 Aug 18;24(9):101055. doi: 10.1016/j.mcpro.2025.101055 (PMC12475854; doi:10.1016/j.mcpro.2025.101055)
Supplement: Supplemental Figures [file mmc1.pdf]

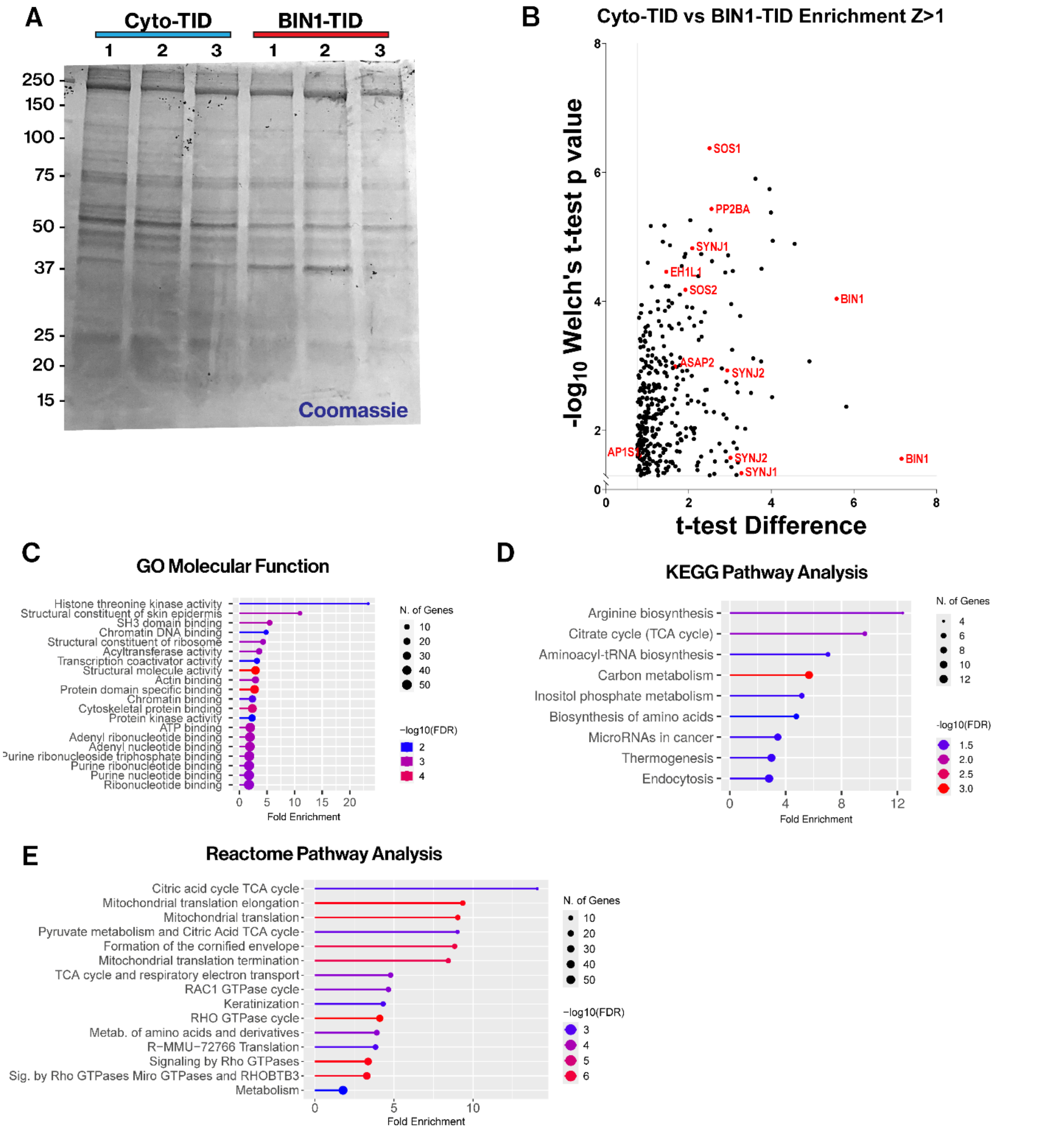

Supplemental Fig. S1. **N2a cells stably expressing BIN1-TID label known BIN1-associated proteins and molecular functions.** *A*, Streptavidin magnetic beads captured biotinylated protein from stable N2a cells stably expressing Cyto-TID and BIN1-TID. Protein eluted from the beads, run on a gel and total protein stained with Coomassie. *B*, Volcano plot displaying the N2a BIN1-TID positive protein hits for z-score >1 and previously reported BIN1-associated proteins labeled in red, t-test difference (x>0.758) vs. -$\log_{10}$ Welch's t-test p-value (y>1.301). *C*, GO molecular function (FDR <0.05), node size (number of genes), node color (-$\log_{10}$FDR) and fold enrichment on the x-axis. *D*, KEGG pathway analysis (FDR <0.05), node size (number of genes), node color (-$\log_{10}$FDR). *E*, Reactome pathway analysis (FDR <0.05), node size (number of genes), node color (-$\log_{10}$FDR).

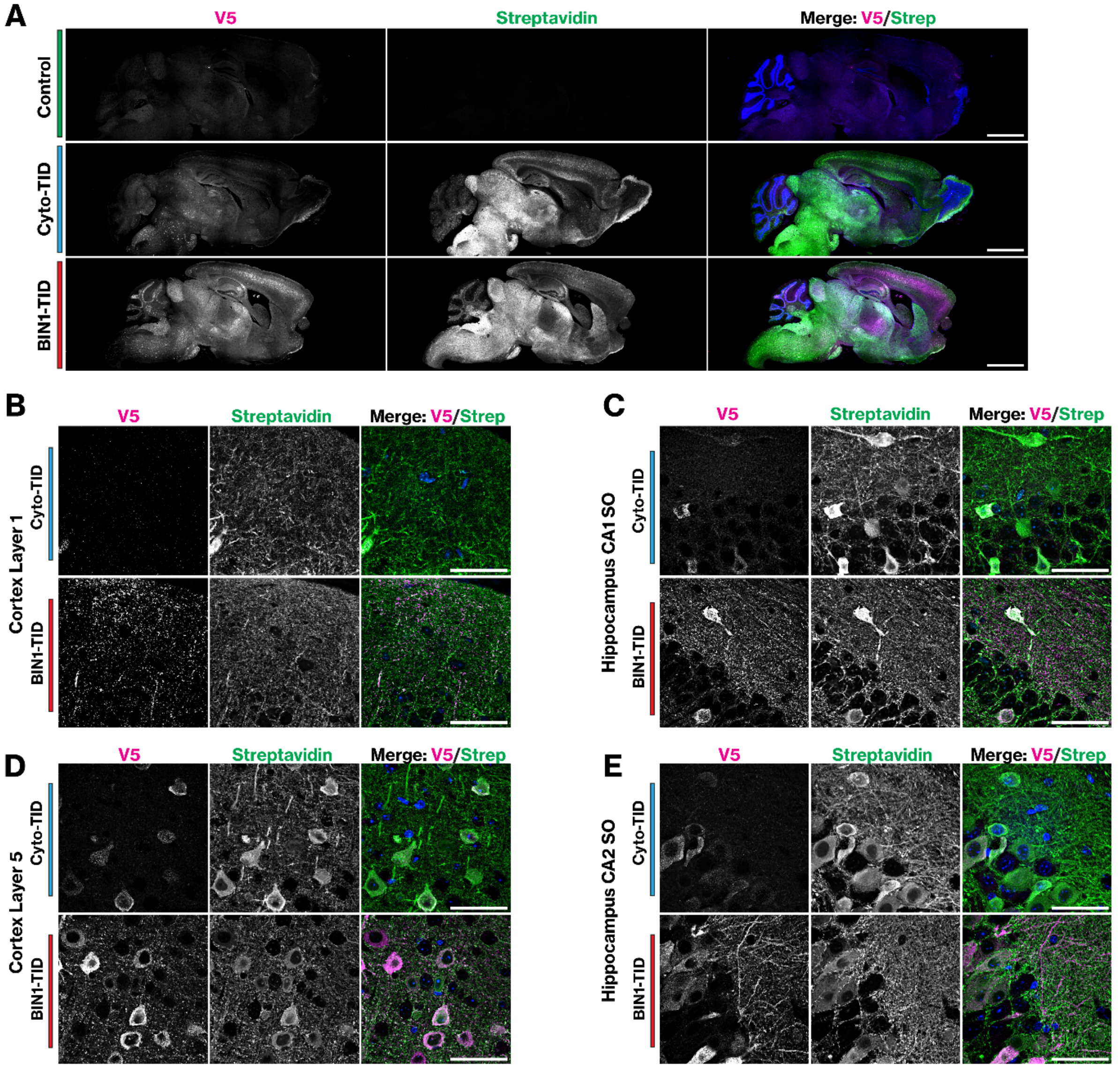

Supplemental Fig. S2. **Robust protein biotinylation in Cyto-TID and BIN1iso1-TID transduced mouse neurons.** *A*, IF of Control (No TID but with biotin treatment), Cyto-TID, and BIN1iso1-TID hemibrains using anti-V5 and Streptavidin. Overlap of images (scale bar = 2 mm). *B*, IF of cortex layer 1 comparing Cyto-TID and BIN1iso1-TID using anti-V5 and streptavidin. Images are 2 µm z-stacks projected as a maximum projection (scale bar = 50 µm). *C*, IF of hippocampus CA1 SO comparing Cyto-TID and BIN1iso1-TID using anti-V5 and streptavidin. Images are 2 µm z-stacks projected as a maximum projection (scale bar = 50 µm). *D*, IF of cortex layer 5 comparing Cyto-TID and BIN1iso1-TID using anti-V5 and streptavidin. Images are 2 µm z-stacks projected as a maximum projection (scale bar = 50 µm). *E*, IF of hippocampus CA2 SO comparing Cyto-TID and BIN1iso1-TID using anti-V5 and streptavidin. Images are 2 µm z-stacks projected as a maximum projection (scale bar = 50 µm).

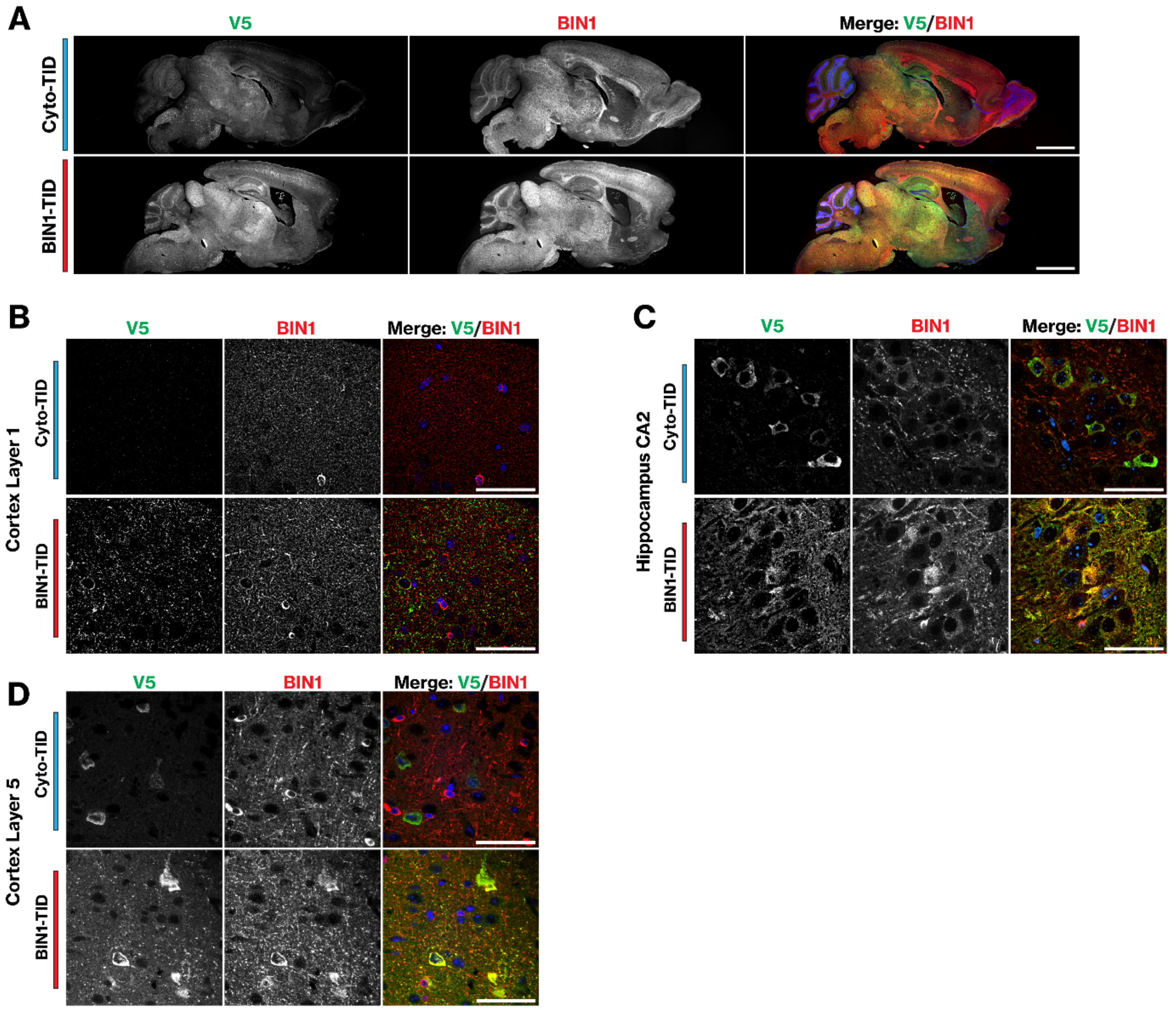

Supplemental Fig. S3. **Overexpression of BIN1iso1-TID overlaps with endogenous mouse BIN1 in mouse brain neurons.** *A*, IF of Cyto-TID, and BIN1iso1-TID hemibrains using anti-V5 and anti-BIN1. Overlap of images (scale bar = 2 mm). *B*, IF of cortex layer 1 comparing Cyto-TID and BIN1iso1-TID using anti-V5 and anti-BIN1. Images are 2 μm z-stacks projected as a maximum projection (scale bar = 50 μm). *C*, IF of hippocampus CA2 comparing Cyto-TID and BIN1iso1-TID using anti-V5 and anti-BIN1. Images are 2 μm z-stacks projected as a maximum projection (scale bar = 50 μm). *D*, IF of cortex layer 5 comparing Cyto-TID and BIN1iso1-TID using anti-V5 and anti-BIN1. Images are 2 μm z-stacks projected as a maximum projection (scale bar = 50 μm).

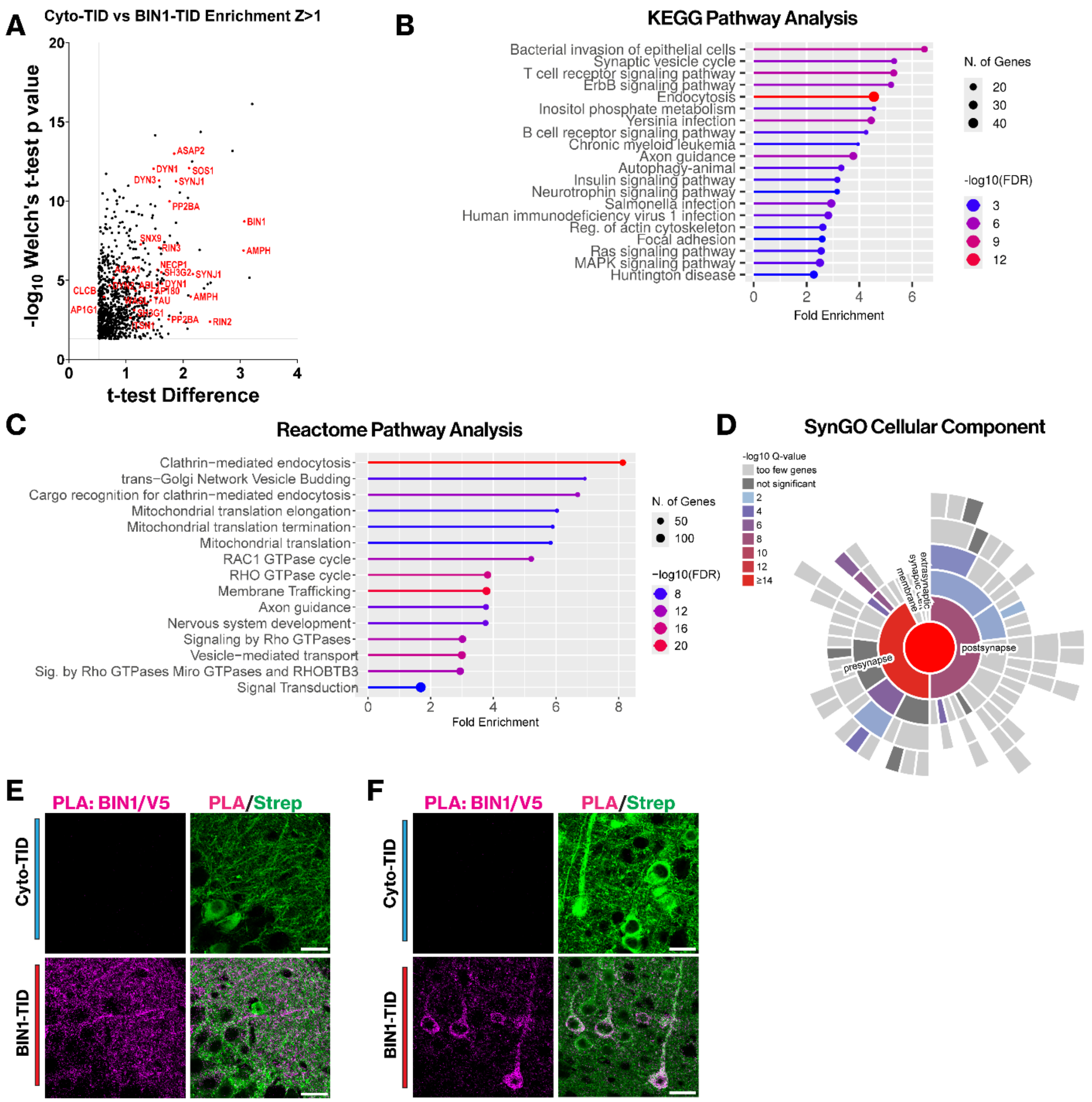

Supplemental Fig. S4. **BIN1-TID colocalizes with endogenous mouse BIN1 and biotinylates presynaptic proteins**. *A*, Volcano plot displaying the mouse brain neuron BIN1-TID positive protein hits for z-score >1 and previously reported BIN1-associated proteins labeled in red, t-test difference (x>0.526) vs. -$\log_{10}$ Welch's t-test p-value (y>1.301). *B*, KEGG pathway analysis (FDR <0.05), node size (number of genes), node color (-$\log_{10}$FDR). *C*, Reactome pathway analysis (FDR <0.05), node size (number of genes), node color (-$\log_{10}$FDR). *D*, SynGO cellular component analysis (Q-value < 0.05) color corresponding to -$\log_{10}$Q-value and annotated with synaptic compartments showing preferential enrichment of the BIN1-TID interactome in the presynaptic terminal. *E*, PLA using primary antibodies against V5 and BIN1 of the hippocampus CA2 SO. Counterstained with streptavidin in green (scale bar = 25 μm). *F*, PLA using primary antibodies against V5 and BIN1 of cortex layer 5. Counterstained with streptavidin in green (scale bar = 25 μm).

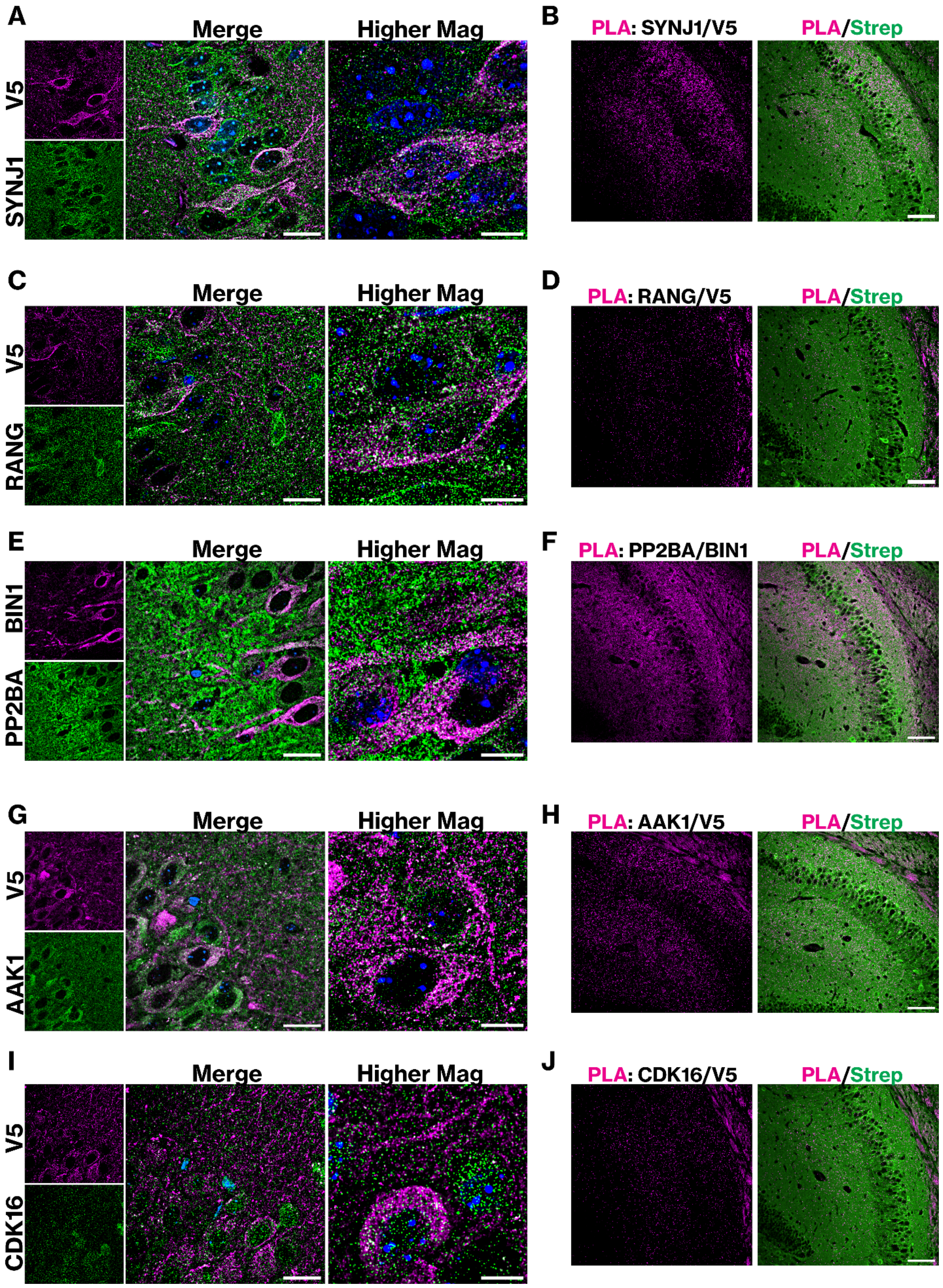

A
Merge
Higher Mag
V5
SYNJ1
B
PLA: SYNJ1/V5
PLA/Strep
C
Merge
Higher Mag
V5
RANG
D
PLA: RANG/V5
PLA/Strep
E
Merge
Higher Mag
BIN1
PP2BA
F
PLA: PP2BA/BIN1
PLA/Strep
G
Merge
Higher Mag
V5
AAK1
H
PLA: AAK1/V5
PLA/Strep
I
Merge
Higher Mag
V5
CDK16
J
PLA: CDK16/V5
PLA/Strep

Supplemental Fig. S5. **BIN1-TID is within a molecular distance of SYNJ1, RANG, PP2BA, AAK1, and CDK16 in the hippocampus.** *A*, IF of BIN1iso1-TID hippocampus using anti-SYNJ1 and anti-V5. Overlap of images (scale bar = 25 µm). Higher magnification images are z-stacks projected as a Sum (scale bar = 10 µm) *B*, PLA using anti-V5 and -SYNJ1. The merge shows PLA signal and streptavidin staining of biotinylated protein for spatial reference (scale bar = 200 µm). *C*, IF of BIN1iso1-TID hippocampus using anti-RANG and anti-V5. Overlap of images (scale bar = 25 µm). Higher magnification images are z-stacks projected as a Sum (scale bar = 10 µm). *D*, PLA of BIN1iso1-TID hippocampus using anti-V5 and anti-RANG. Co-stained with streptavidin to display biotinylated proteins (scale bar = 200 µm). *E*, IF of BIN1iso1-TID hippocampus using anti-PP2BA and anti-BIN1. Overlap of images (scale bar = 25 µm). Higher magnification images are z-stacks projected as a Sum (scale bar = 10 µm). *F*, PLA of BIN1iso1-TID using anti-PP2BA and anti-BIN1. Co-stained with streptavidin to display biotinylated proteins for reference (scale bar = 200 µm). *G*, IF of BIN1iso1-TID hippocampus using anti-AAK1 and anti-V5. Overlap of low mag images (scale bar = 25 µm). Higher magnification images are z-stacks projected as a Sum (scale bar = 10 µm). *H*, PLA using anti-AAK1 and anti-V5 antibodies. Co-stained with streptavidin to display biotinylated proteins for reference (scale bar = 200 µm). *I*, IF of BIN1iso1-TID hippocampus using anti-CDK16 and anti-V5 antibodies. Overlap of low mag images (scale bar = 25 µm). Higher magnification images are z-stacks projected as a Sum (scale bar = 10 µm). *J*, PLA of BIN1iso1-TID using anti-CDK16 and anti-V5 antibodies. Co-stained with streptavidin to display biotinylated proteins for reference (scale bar = 200 µm).
